# Meiotic Transmission of an In Vitro*–*Assembled Autonomous Maize Minichromosome

**DOI:** 10.1371/journal.pgen.0030179

**Published:** 2007-10-19

**Authors:** Shawn R Carlson, Gary W Rudgers, Helge Zieler, Jennifer M Mach, Song Luo, Eric Grunden, Cheryl Krol, Gregory P Copenhaver, Daphne Preuss

**Affiliations:** 1 Chromatin, Chicago, Illinois, United States of America; 2 Department of Biology, The University of North Carolina at Chapel Hill, Chapel Hill, North Carolina, United States of America; 3 Carolina Center for Genome Sciences, The University of North Carolina at Chapel Hill, Chapel Hill, North Carolina, United States of America; 4 Department of Molecular Genetics and Cell Biology, The University of Chicago, Chicago, Illinois, United States of America; The Salk Institute for Biological Studies, United States of America

## Abstract

Autonomous chromosomes are generated in yeast (yeast artificial chromosomes) and human fibrosarcoma cells (human artificial chromosomes) by introducing purified DNA fragments that nucleate a kinetochore, replicate, and segregate to daughter cells. These autonomous minichromosomes are convenient for manipulating and delivering DNA segments containing multiple genes. In contrast, commercial production of transgenic crops relies on methods that integrate one or a few genes into host chromosomes; extensive screening to identify insertions with the desired expression level, copy number, structure, and genomic location; and long breeding programs to produce varieties that carry multiple transgenes. As a step toward improving transgenic crop production, we report the development of autonomous maize minichromosomes (MMCs). We constructed circular MMCs by combining *DsRed* and *nptII* marker genes with 7–190 kb of genomic maize DNA fragments containing satellites, retroelements, and/or other repeats commonly found in centromeres and using particle bombardment to deliver these constructs into embryogenic maize tissue. We selected transformed cells, regenerated plants, and propagated their progeny for multiple generations in the absence of selection. Fluorescent in situ hybridization and segregation analysis demonstrated that autonomous MMCs can be mitotically and meiotically maintained. The MMC described here showed meiotic segregation ratios approaching Mendelian inheritance: 93% transmission as a disome (100% expected), 39% transmission as a monosome crossed to wild type (50% expected), and 59% transmission in self crosses (75% expected). The fluorescent *DsRed* reporter gene on the MMC was expressed through four generations, and Southern blot analysis indicated the encoded genes were intact. This novel approach for plant transformation can facilitate crop biotechnology by (i) combining several trait genes on a single DNA fragment, (ii) arranging genes in a defined sequence context for more consistent gene expression, and (iii) providing an independent linkage group that can be rapidly introgressed into various germplasms.

## Author Summary

The production of transgenic maize has traditionally used techniques that integrate DNA fragments into a host chromosome. This can disrupt important native genes or can lead to poor expression of the added gene; consequently, large numbers of transgenic plants must be screened to find one suitable for commercial use. Further, there is a limit to the amount of DNA that can be integrated, making it difficult to add multiple genes at one time. Here, we describe a new system for delivering genes to maize. We constructed a minichromosome vector that remains separate, or autonomous, from the plant's chromosomes when introduced into maize cells. These minichromosomes were constructed from DNA sequences that naturally occur in maize centromeres, the chromosomal regions needed for inheritance. We characterized the behavior of Maize Minichromosome 1 (MMC1) through four generations, showing that it is efficiently inherited and that the genes it carries are expressed. This work makes it possible to design minichromosomes that carry several genes, enhancing the ability to engineer plant processes, including improving disease resistance, drought tolerance, or the production of complex biochemicals.

## Introduction

Agricultural crops have the potential to meet escalating global demands for affordable and sustainable production of food, fuels, therapeutics, and biomaterials [1]. While standard integrative plant transformation can often meet these needs by safely introducing novel genes into plant chromosomes, they are limited in efficiency. Typically, biological delivery of DNA carried on an *Agrobacterium* T-DNA plasmid, or biolistic delivery of small DNA-coated particles is employed to transfer and integrate desired genes into a host plant chromosome [2]. Integration at random sites results in unpredictable transgene expression due to position effect variegation, variable copy number from tandem integrations, and frequent loss of gene integrity as a result of unpredictable breakage and end joining [2,3]. For highly characterized crops such as maize, transgene integration can also result in genetic linkage of the introduced genes to portions of the genome known to encode loci that confer undesired phenotypes, adding complexity when the transgenic locus is introgressed into other varieties [4,5]. Recent advances in gene integration technologies have aimed to surmount some of these difficulties. For example, zinc finger–mediated homologous recombination or site-specific recombination could eliminate the unpredictable expression that results from random insertion into the plant genome [6,7]. In addition, combining binary T-DNA elements with bacterial artificial chromosome (BAC) technology to produce BiBACs has the potential to introduce larger DNA fragments into the host genome [8,9]. In contrast to these systems, the maize minichromosomes described here remain separate from the host chromosomes, and thus provide an alternative approach with important benefits. Indeed, although precise integration into host chromosomes has long been a routine technique in Saccharomyces cerevisiae, the facile properties of autonomous vectors often make them a preferred choice for numerous applications, including commercial-scale protein production.

The first eukaryotic minichromosomes employed a simple centromere (CEN) sequence from the budding yeast S. cerevisiae, incorporated into versatile circular CEN and linear yeast artificial chromosome (YAC) vectors [10,11]. These yeast vectors were used to define a 125-bp DNA fragment sufficient for mitotic and meiotic centromere function [12]. While circular CEN vectors are most useful for carrying smaller DNA fragments, YAC vectors can carry megabase quantities of DNA and are convenient for manipulating large fragments of DNA [13]. Similarly, with carrying capacities of hundreds of kb, human artificial chromosomes (HACs) provide advantages over other in vitro*–*assembled vectors used in human cell transfection [14]. HACs containing tandem repeats of a 171-bp alpha satellite sequence can be maintained either as circular or linear, telomere-containing, episomes [15–19].

DNA sequences that can form stable minichromosomes are able to recapitulate centromere functions de novo by recruiting essential DNA binding proteins and epigenetic modifications. In human cells, different satellite arrays vary in their ability to efficiently form HACs, based on their satellite monomer sequence, chromosomal origin, array length, higher-order structure, and even vector composition [20–23]. These DNA sequences recruit centromere binding protein A (CENP-A), which substitutes for histone H3 to form centromeric nucleosomes; this protein marks active centromeres in S. cerevisiae (Cse4p), Schizosaccharomyces pombe (Cnp1), Drosophila melanogaster (Cid), Arabidopsis thaliana (*HTR12*), Zea mays (CENH3), and Homo sapiens (CENP-A) [24–29]. CENP-A complexes are maintained through mitosis and meiosis [30], resulting in an epigenetic mark that may be more important in perpetuating centromere activity than the underlying DNA sequence. Evidence for this role in centromere maintenance comes from human neocentromeres [31], where, at a very low frequency, ectopic centromeres are nucleated in regions that lack satellite DNA. Once formed, these neocentromeres are efficiently perpetuated. The ability to form centromeres on naked DNA also depends on cell type in mammalian systems; indeed, HAC formation has only been demonstrated in HT1080 fibrosarcoma cells. Yet once established, HACs can be transferred to other mammalian cell types, where they are stably maintained [32].

Maize centromeres contain repetitive sequences that are similar to those found in mammalian centromeres; for example, analogous to the tandem arrays of alpha satellite found in human centromeres, large tandem arrays of the 156-bp maize CentC satellite bind to CENP-A [33,34,28]. These satellite arrays are often interrupted by CRM, a centromere-specific retroelement that also binds CENP-A [28]; the significance of this arrangement for centromere function is unknown. Some maize varieties also have supernumerary B chromosomes with a distinct centromere satellite sequence, ZmBs [35,36]. These B chromosomes lack essential genes, and thus have been particularly useful for discerning the relationship between centromere structure and meiotic transmission [37–39]. A series of deletion derivatives of natural B chromosomes, derived from an A-B translocation event, showed a strong dependence on centromere size—the smallest functional derivative contained a 110-kb centromere and resulted in a meiotic transmission rate of 5%, yet showed a high stability in mitosis [39]. More recently, telomere-mediated chromosomal truncation was used to generate deletion derivatives from both A and B maize chromosomes [40]. Transgenes carried on these derivative chromosomes (or “engineered minichromosomes”) were expressed and meiotic inheritance ranged from 12% to 39% [40]. While this telomere-truncation approach can deliver both transgenes and sequences that promote site-directed integration, its utility for commercial applications may be limited—most commercial maize hybrids lack B chromosomes, and the duplications needed to maintain truncated A chromosomes may prove challenging for regulatory approval.

As described below, we developed autonomous minichromosomes that do not rely on alteration of endogenous chromosomes. We constructed plasmids carrying maize centromeric repeats, delivered purified constructs to embryogenic maize tissue, and assessed their ability to promote the formation of MMCs. MMC1 was characterized in detail; this CentC-based construct contained 19 kb of centromeric DNA and conferred efficient mitotic and meiotic inheritance through at least four generations when introduced into plant cells. This approach could be widely used in commercial corn production—a construct with a defined sequence will facilitate regulatory review, while MMC independence from the host genome reduces the risk of alternations that impair host fitness.

## Results

### Screen for Functional Maize Minichromosomes

We probed a maize genomic BAC library with repetitive sequences, including those typically found in maize centromeres (Materials and Methods; Table 1). Clones enriched in satellite sequences, centromeric retroelements, and other repetitive sequences were chosen to assess whether they can form MMCs when delivered to plant cells*.* While our study did not explore the interactions between MMC DNA inserts and kinetochore or spindle proteins, we hereafter refer to these fragments as “centromeric,” based on the typical genomic location of the sequences they contain. In vitro Cre-lox recombination was used to fuse selected BAC clones to a circular vector containing a plant selectable marker (*nptII*) and a cell-autonomous reporter gene (nuclear-expressed *DsRed*), forming circular constructs. While circular and linear HACs containing repetitive centromeric DNA are mitotically transmitted in human cell lines, circular HACs can confer higher levels of meiotic transmission in transgenic mice [3,20,32]. Consequently, we focused our initial efforts on circular constructs, generating plants by bombarding embryogenic maize tissue with purified candidate MMC DNA, selecting transformed cells expressing the *nptII* marker and resistant to antibiotics, and propagating regenerated plants in the absence of selection (see Materials and Methods). Of the 102 constructs bombarded, 66 gave rise to regenerated plants; 52 of these constructs were randomly chosen and characterized as described below.

**Table 1:**
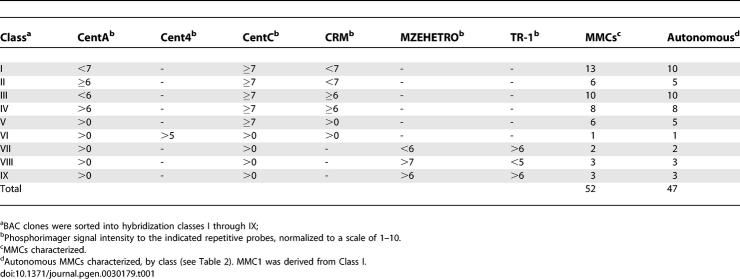
Classification of Maize BAC Clones Containing Repetitive DNA

**Table 2:**
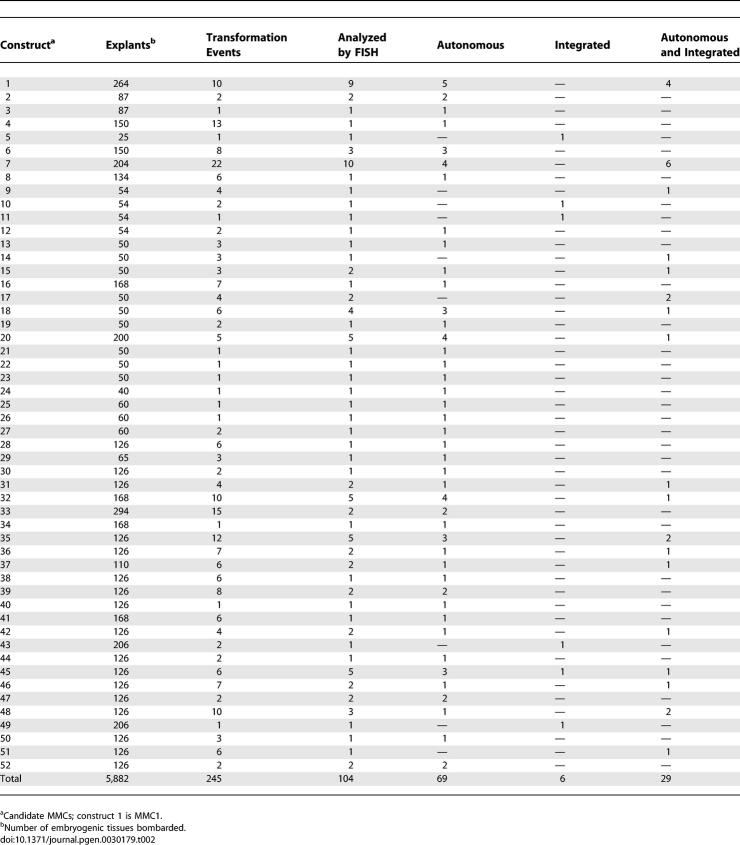
MMC Transformation Events and FISH Analysis

**Figure 1:**
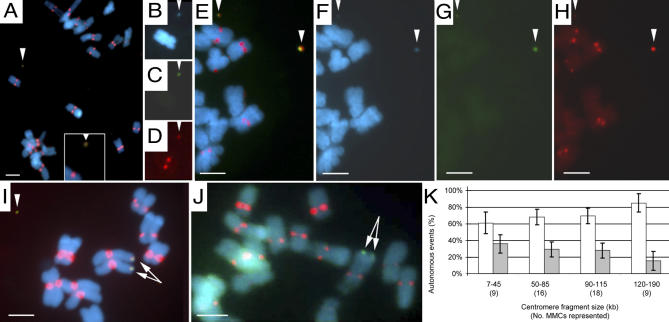
Generation of Autonomous Minichromosomes (A–H) Metaphase chromosome spreads from MMC1 event V-1: (A–D) T1 plant; (B–D) correspond to the region denoted by the arrowhead in (A); (E–H) T2 plant. DNA is stained with DAPI ([B F], blue) and labeled with FISH probes specific for the *DsRed* and *nptII* gene cassette ([C, G], green); or centromere sequences ([D, H], red). (I, J) Event V-4 with autonomous and integrated copies of MMC1 (I); pCHR758 (noncentromeric control) (J). Autonomous minichromosomes (arrowheads); integrated constructs appear as pairs of FISH signals (arrows); size bar, 5 μm. (K) Centromere fragments across a wide size range enable autonomous MMC inheritance. For each size category, the percentage of transformation events (total = 52) that yielded only an autonomous MMC (white bars) or both an autonomous and integrated MMC in the same cell (grey bars) is shown; the number of MMCs in each category is noted parenthetically; error bars indicate standard error.

To evaluate whether the introduced constructs were maintained autonomously or instead had integrated into the genome, we preformed fluorescence in situ hybridization (FISH). We arrested root tip cells in mitosis, and stained chromosome spreads [41] with rhodamine and fluorescein-labeled probes corresponding to centromeric repeats and to MMC-encoded genes, respectively (Figure 1A–1I). FISH labeling of integrated control constructs resulted in adjacent pairs of metaphase FISH signals corresponding to replicated sister chromatids (Figure 1J). While some MMC constructs integrated (see below), we considered MMCs autonomous when (i) ≥70% of the cells examined (*n* ≥ 15) contained signals that were clearly distinct from the DAPI-stained host chromosomes, (ii) integrated signals were not detected, and iii) the fluorescent probe corresponding to the MMC-encoded genes colocalized with the probe to repetitive centromeric DNA, suggesting an intact construct and making it unlikely that the signal was due to noise. In many cases, the detection of a DAPI signal that colocalized with the FISH probes provided further evidence of MMC autonomy (Figure 1B–1D and 1F–1H).

Based on these criteria, 47/52 (90%) of the constructs we evaluated with FISH were able to form an autonomous MMC, and 43/52 (with centromeric inserts ranging in size from 7 to 190 kb) gave rise to plants that contained only an autonomous MMC (Table 2). This unexpectedly high rate of recovering autonomous MMCs suggests that embryogenic maize tissue readily establishes MMCs from purified DNA and that the BAC clones that yielded transformed plants contained sequences that efficiently promote MMC formation. The efficiency of forming an autonomous MMC increased slightly, although not significantly (*t*-test), as the size of the genomic DNA insert increased (Figure 1K). A similar analysis of human centromeric fragments showed that as little as 35 kb could generate a HAC, while larger fragments (70–220 kb) were required for efficient HAC formation [42]. As described below, MMCs were often efficiently inherited; nonetheless, MMC integration was detected only during the initial transformation event, and not in subsequent generations (T1 through T4, 0/312 metaphase spreads, 33 plants). Below, we report on the composition and behavior of one of the MMC constructs (MMC1) in detail.

### Meiotic Inheritance of MMC1

**Figure 2:**
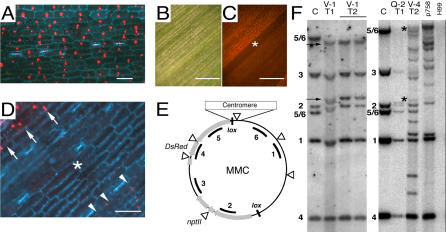
MMC Gene Expression and Structure (A) Fluorescent detection of nuclear-localized *DsRed* in MMC1 maize leaf; size bar, 50 μm. (B, C) Detection of *DsRed* sectors in a T2 plant leaf from event V-1 under (B) bright-field and (C) fluorescence microscopy; size bars, 0.5 mm. (D) high magnification view of image shown in (C) with the corresponding sector, comprising all cell layers, indicated by an asterisk; the edge of a sector that comprises only the adaxial cell layer is indicated by arrowheads, cells with typical *DsRed* expression are indicated by arrows. Size bar, 50 μm. (E) MMC consisting of a pCHR758 backbone and a centromere-derived insert, gene expression cassettes (grey), centromeric inserts (box), BglII restriction sites (arrowheads), and probes used for FISH and Southern blot analyses are indicated. (F) Southern blot of DNA digested with BglII and hybridized to probes 1–6 (E); Bands 1–4 measure 2,067, 3,167, 5,227 and 790 bp, respectively; those hybridizing to probes 5 and 6 vary in size, depending on the location of BglII sites within the centromeric DNA insert. MMC1 Control (c, lanes 1 and 5) DNA was purified from E. coli and hybridization patterns were compared to DNA from plant cell extracts derived from MMC1 events V-1 (lanes 2–4), Q-2 (lane 6), and V-4 (lane 7), as well as from plants transformed with pCHR758 (lane 8) and untransformed wild type (H99, lane 9). For events V-1 and Q-2, bands differing from bacterial grown controls are indicated (arrows and asterisk, respectively).

Control transformations performed with a *DsRed/nptII* plasmid lacking a centromere-derived insert (pCHR758) contained a construct that integrated, as expected, into a native chromosome (7/7 events, Figure 1J). In contrast, for MMC1, 5/9 independent transformation events yielded solely an autonomous chromosome (Figure 1A–1H, see also Figure S1) and 4/9 generated both integrated and autonomous copies (Figure 1I). We tested the ability of these MMCs to confer inheritance by crossing T0 transformants to wild type, growing the progeny without selection, and monitoring nuclear-localized *DsRed* fluorescence (Figure 2A). Because we typically observed only one MMC per cell (monosomic), we expected these T0 plants to behave as hemizygotes; if the MMC obeyed Mendelian inheritance, then such crosses would yield *DsRed* progeny in a 1:1 ratio. Ten T0 plants (derived from three events) carrying solely an autonomous MMC1 copy were crossed to wild-type pollen. Two of the MMC1 events (V-1 and Q-1) indeed transmitted *DsRed* to T1 offspring in ratios that did not differ significantly from Mendelian predictions (Table 3). However, for a third MMC1 event (Q-2), we saw a significant reduction in *DsRed^+^* progeny compared to expectations (52%, Table 3), suggesting genetic instability. PCR analysis of the progeny from this cross confirmed that the plants lacking *DsRed* expression also lacked *DsRed* sequences, indicating that the deviation from Mendelian assortment was not due to silencing of gene expression. Instead, the elevated MMC loss rate in this event could result from in planta modifications of the centromeric insert or from epigenetic effects that led to less robust segregation [43]. As expected, performing a similar analysis of six events carrying an integrated pCHR758 backbone yielded Mendelian inheritance ratios (118:119 *DsRed* ^+^:*DsRed* ^−^; *p >* 0.05).

**Table 3:**
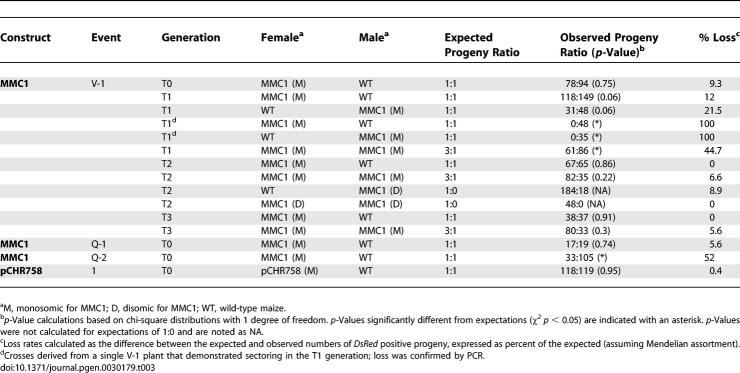
Meiotic Inheritance of MMC1

FISH analysis showed that T1 plants from event V-1 retained an autonomous MMC: a *DsRed*-containing episome was present in 80% of root metaphase cells (*n* = 44), a detection level consistent with previous artificial chromosome studies [44]. Because we consistently observed *DsRed* expression in nearly every cell from these plants (see below), we conclude that the absence of an MMC FISH signal in 20% of root cells likely represents the challenges of retaining and detecting every MMC throughout the FISH protocol. To monitor MMC1 inheritance in subsequent generations and through both male and female gametes, we performed a series of crosses with T1, T2, and T3 plants derived from event V-1 and monitored *DsRed* transmission. When male or female monosomic MMC1 plants were crossed to wild type, *DsRed* segregation was not significantly different from Mendelian inheritance ratios (1:1, Table 3). For one exceptional T1 plant, however, such crosses yielded no progeny containing MMC1 (female: 0:48, male: 0:35; Table 3); the absence of *DsRed*-encoding DNA in these progeny was confirmed by PCR, supporting the view that this MMC was indeed autonomous. Interestingly, the leaf tissue of this plant had prominent mitotic *DsRed* leaf sectors, suggesting a high rate of MMC instability.

When we self-pollinated T2 and T3 hemizygous plants derived from event V-1, we observed *DsRed^+^* inheritance in a ratio that did not significantly differ from a 3:1 Mendelian pattern. However, in a second case of non-Mendelian assortment, a self-cross in the T1 generation yielded a 1:1 *DsRed^+^* inheritance ratio, suggesting loss of MMC1 from either the male or female floral tissue. Nonetheless, this cross was useful for generating plants that potentially carried two copies of MMC1 (homozygous disomes). Crossing pollen from a candidate T2 disome onto five different maize inbreds yielded 184 *DsRed^+^*:18 *DsRed* ^−^ offspring (*p >* 0.05 for disomy). Similarly, self-pollinating potentially disomic T2 or T3 plants produced 48:0 and 24:0 *DsRed^+^*:*DsRed* ^−^ offspring, respectively. Quantitative PCR (qPCR) analysis of the potentially disomic T2 plants confirmed 2.00 and 1.90 (standard error = 0.08) *DsRed* copies per cell, respectively (see Materials and Methods)*.*

### MMC1 Stability: Gene Expression and Structure

For most plants carrying an autonomous MMC, nuclear *DsRed* expression was observed in nearly every leaf cell, indicating stability through mitosis. In some cases, however, sectors that lacked *DsRed* expression were found (Figure 2B–2D); these were generally limited to a few cell files. In reproductive tissues, such sectors could be responsible for the aberrant meiotic MMC segregation described above. In total, mitotic sectors of *DsRed* expression from MMC1 were detected in 3.6% of T0 plants (*n* = 56), 3.0% of T1 plants (*n* = 404), 1.9% of T2 plants (*n* = 837), and no T3 (*n* = 738) or T4 (*n* = 250) plants. The reduced sectoring frequency as plants advanced through generations suggests a gradual increase in MMC stability due to changes in DNA composition, epigenetic modifications, or MMC copy number in mitotic cells. A similar stabilization through generations was observed in an oat-maize addition line [45]. We also found that 60 d of crowding and drought stress did not appreciably alter MMC1 stability; *DsRed* expression was found in every T2 and T3 plant from event V-1 grown under stress (151 and 159 plants, respectively). Moreover, pollen from stressed hemizygous T2 plants demonstrated Mendelian *DsRed* segregation (281:238 *DsRed* ^+^:*DsRed* ^−^; *p >* 0.05).

To assess the structure of MMC1 through generations, we performed Southern blot analysis, probing to detect all of the unique sequence bands contained in the MMC construct (Figure 2E and 2F). MMC structural alterations sometimes occurred during transformation, often involving the centromeric insert, rather than the gene cassette (Figure 2F). Additional rearrangements were typically not detected after the T1 generation (*n* = 5), although the repetitive nature of the centromeric fragment made it impossible to thoroughly evaluate its structure on these blots. In addition, Southern blot analysis showed centromeric alterations in event V-1 that were transmitted from the T1 parent to the T2 progeny. Event Q-2 suffered a larger alteration of the centromeric fragments (indicated by an asterisk in Figure 2F), potentially explaining its reduced meiotic stability. In contrast, an event carrying both integrated and autonomous MMC1 copies (V-4) showed a more complicated pattern, as did plants carrying integrated pCHR758. As expected for independently assorting loci, when plants from event V-4 were crossed to wild type, the autonomous and integrated copies segregated: FISH evaluation of *DsRed*-expressing T2 plants yielded a 1:4:2 ratio (autonomous:autonomous and integrated:integrated).

### MMC1 Composition

**Figure 3:**
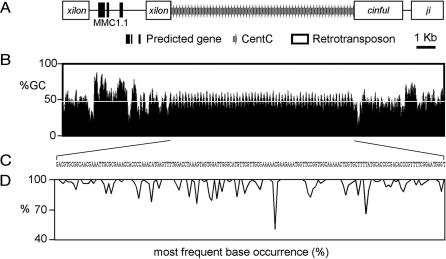
Sequence Analysis of the MMC1 Centromeric Fragment (A) Assembled sequence. (B) Fifty base-pair sliding window analysis of MMC1 GC content; white line, genomic average = 49.5%. (C) Consensus sequence of ∼60 MMC1 CentC repeats. (D) The occurrence of the most frequent base (%) for each nucleotide in the CentC consensus.

MMC1 was originally identified by its strong hybridization to a CentC probe, suggesting it contained a high percentage of this satellite repeat (Table 1). Sequence analysis confirmed the presence of CentC repeats arranged in an uninterrupted tandem array (GenBank accession number in Supporting Information; Figure 3A and 3B). The repetitive nature of CentC made a precise assembly of this array challenging; we used rare DNA polymorphisms within the repeats to aid in sequence assembly, and confirmed the overall length of the array (approximately 9 kb) with restriction enzyme digestion and gel electrophoresis. Based on these measurements and quantitative dot blot hybridization (see Materials and Methods) the CentC array contains between 59 and 64 (61.4 ± 2.3) copies. CentC repeat alignments showed that each base is conserved at an average frequency of 96.1% (Figure 3C and 3D), a level consistent with previously reported plant satellite conservation [46]. Clustering algorithms failed to detect higher order repeat patterns in MMC1 (unpublished data).

While the maize genome has an average GC content of 49.5%, the 5.6- and 4.8-kb regions flanking the CentC array of MMC1 reach 88% and 70% GC, respectively (Figure 3B). Overall, the GC content of the MMC1 centromeric insert is 48%; by comparison, published sequences from two maize centromeric BACs had 43% and 47% GC content [34] while *Arabidopsis* and rice centromere DNA averages 35%–40% and 39%–48%, respectively [47,48]. MMC1 encodes four regions with similarity to retrotransposons *xilon*, *cinful*, or *ji* [49], as well as a 453-bp open reading frame (MMC1.1) that potentially encodes a novel protein of unknown function, complete with a promoter and poly-A signal (Figure 3A). BLAST searches of GenBank revealed no evidence for MMC1.1 expression, but transcripts >95% identical to CentC and to the MMC1 retrotransposons were abundant. Transcription of centromeric repeats is important for centromere function in S. pombe [50], and *Arabidopsis* satellites are also transcribed [51]. The centromere-specific histone CENH3 binds to transcripts corresponding to CentC and to the retrotransposon CRM, suggesting a role for these RNAs in centromere function [52]; it is not clear if *xilon*, *cinful*, or *ji* transcripts play a similar role. Retrotransposons also can nucleate the formation of heterochromatin that can spread to nearby regions [53], although MMC1-encoded *DsRed* and *nptII* were readily expressed, despite their separation of 3.3 and 6.2 kb, respectively, from retrotransposons.

## Discussion

Taken together, the experiments described above strongly support the conclusion that MMC1 can be maintained as an autonomous chromosome: it remains distinct from host chromosomes, its gene cassette is structurally stable through at least four generations, the genes it carries are expressed and transmitted through meiosis and mitosis, and, in some cases, it can be lost from the genome at a frequency higher than that of a native chromosome. Interestingly, classical studies of plant trisomics typically reveal far greater defects in meiotic inheritance [54], while inheritance levels similar to those we observed with MMCs have been reported in other artificial chromosome systems. For example, a monosomic mouse artificial chromosome that showed <1% mitotic loss when carried in human, bovine, or mouse cell lines [55], suffered only 4% meiotic loss through the mouse germline [44]. Furthermore, while classically studied ring chromosomes are often unstable [56], circular MMC inheritance through four generations was reminiscent of that observed for circular chromosomes from yeast [57], mammals [32], and maize [58]. These data suggest that this MMC could be maintained indefinitely.

MMC centromere sequences, like those that make up endogenous centromeres, could rely on the kinetochore and spindle machinery for faithful segregation, or could be inherited through alternative mechanisms. For example, in plants, dense heterochromatic domains known as knobs or neocentromeres migrate to daughter cells by moving along the sides of the spindle, rather than by kinetochore-mediated association with the ends of microtubules. This process results in preferential assortment to gametes, and consequently greater than expected inheritance ratios (termed meiotic drive) [59]. Heterochromatin-based mechanisms of assortment have also been characterized in *Drosophila*, where chromosomes that lack evidence of meiotic exchange (chiasmata) are nonetheless inherited at Mendelian ratios [60]. Further, in S. cerevisiae, which lacks appreciable heterochromatin, the 2-μm circle plasmid is partitioned at an efficiency that rivals that of yeast chromosomes; this assortment relies on microtubule-mediated attachment of cohesin to 2 μm of DNA [61]. The possibility that MMC segregation might rely on alternative mechanisms is intriguing; indeed, the relatively small MMCs may differ from mammalian artificial chromosomes in which large alpha satellite arrays bind essential centromere proteins.

Epigenetic factors have been postulated to play a principal role in establishing higher eukaryotic centromeres [43], with studies of human neocentromeres [62] and *Drosophila* strains overexpressing CENH3 [63] suggesting a lack of dependence on specific DNA sequences. On the other hand, HACs are able to efficiently nucleate centromere activity in a sequence-dependent manner, and HAC sequences tend to expand in vivo [20], suggesting a selection for a preferred size and/or composition. The MMC1 DNA that we delivered to plant cells was purified from E. coli and thus lacked eukaryotic epigenetic marks, yet it formed autonomous chromosomes. This MMC construct contained only a 19-kb centromeric insert and is thus substantially smaller than the centromeric regions that were previously known to provide mitotic and meiotic inheritance. For example, the fully sequenced centromere of rice Chromosome 8 contains a satellite array measuring 69 kb [64], and a deletion derivative of the maize B chromosome that measures 110 kb is sufficient to confer meiotic inheritance, albeit inefficiently [39]. While HACs routinely expand to a larger size in vivo, we did not detect major rearrangements or expansions of MMC1 DNA through two plant generations, suggesting that its composition was adequate to establish a minichromosome. Nonetheless, our analysis was unable to fully assess the structure of the repetitive centromeric DNA, and it remains possible that these regions could expand, contract, or rearrange in some other manner.

While the total size of MMC1 is quite small (35 kb), other MMCs, some measuring over 200 kb, were successfully delivered to plants and transmitted through meiosis (unpublished data). This suggests that MMC1 has the capacity to serve as a platform to carry a large number of genes. As this MMC is optimized to commercial performance levels, it will provide an unprecedented opportunity to deliver gene combinations (“stacks”) that confer valuable traits to corn varieties. Long breeding programs are often required to introgress an integrated transgene into desired germplasm, while eliminating undesirable linked loci. Because an MMC forms an independent linkage group, these programs could be accelerated, allowing products to appear in the marketplace sooner. Moreover, the performance and expression of transgenic traits will likely become more predictable and reliable as MMC design rules are understood. Extensions of this minichromosome technology beyond traditional agriculture may enable the construction of multigene pathways to produce pharmaceuticals and other industrial products in plants.

## Materials and Methods

### Construction of candidate MMCs.

A BAC library was created in pBeloBAC11 using MboI-digested DNA from the maize inbred B73. This library was arrayed on nitrocellulose filters and probed separately with repetitive sequences from maize that are often found in centromeres or neocentromeres: CentA, Cent4, CentC, CRM, MZEHETRO, and TR-1; ^32^P-labeled probes were hybridized for 14 h at 65 °C and washed with 0.5× SSC, 1% SDS three times at 65 °C. To identify clones carrying centromere DNA, phosphorimager scans of each hybridization experiment were digitally assembled into a MySQL database. BAC clones with strong hybridization signals to one or more of the repetitive sequences were selected for minichromosome construction (Table 1). First, a high copy number plasmid (pCHR758) carrying the *Arabidopsis* UBQ10 promoter to *DsRed* (Clonetech) and the yeast YAT1 promoter fused to *nptII* was constructed. An 8.5-kb fragment encoding the *DsRed* and *nptII* expression constructs (and lacking a bacterial origin) was liberated from pCHR758 with I-PpoI, purified from an agarose gel (QIAquick Gel Extraction Kit, Qiagen), and circularized by Cre-mediated exchange (New England Biolabs) at two *loxP* sites that flanked the gene expression cassette. BAC clones carrying putative centromere DNA insertions were recombined with this vector via the *loxP* site in pBeloBAC11, generating circular candidate MMC constructs (Figure 2E). These constructs were maintained in E. coli DH10B (Invitrogen).

### Delivery and propagation of candidate MMCs in plants.

MMC constructs grown in E. coli were purified using alkaline lysis or cesium chloride protocols and delivered to embryogenic H99 maize tissues by biolistic bombardment of DNA-coated gold particles as described [65]. Transformed events were identified by selection on Chu's N6 medium containing G418 sulfate (PhytoTechnology Laboratories) or paromomycin (Sigma) and regenerated. Transformed plants were subsequently grown without selection in a soilless mix (Sunshine LC1) in a greenhouse (16-h d, 26–28 °C). Seedlings were grown in 48-well flats (2 sq ft) with one plant per well to the V3 developmental stage and then transplanted into 1.6-gallon pots containing 1:1:1 soil:peat:perlite and grown to maturity. Plants subjected to stress conditions were maintained in 48-well flats for 60 d with watering limited to once per day. MMC containing plants have been advanced through four generations by backcrossing to H99, outcrossing to public maize inbreds, and by self pollination or sibling mating.

### Fluorescence assays.

For *DsRed* expression, leaf 3 (V2 stage of development) was sampled across its entire width (minimally 2,500 cells per sample) and fluorescence was detected using a Zeiss SV-11 dissecting microscope equipped with a rhodamine filter cube (excitation: D540/25; dichroic 565LP; emission: D605/55). Background autofluorescence was detected with a GFP filter cube (excitation: BP 470/40; beamsplitter: FT495; emission: BP 525/50); bona fide *DsRed* fluorescence was not detectable at this excitation wavelength. *DsRed* expression in pollen was determined after fixing florets in 95% ethanol; aceto-carmine staining was subsequently used to assess pollen viability. For FISH, root tips were collected approximately 10 d after transplanting regenerated T0 plants to soil or after germination (T1 through T4 plants). Sampled roots (3–6 per plant) were moistened and exposed to nitrous oxide at 150 psi for 2.5 h to arrest chromosomes in metaphase [66]. Roots were fixed in 90% acetic acid and spread onto poly-lysine coated glass slides by squashing thin cross sections. FISH was performed essentially as described [41] using probes labeled with Alexa488 (pCHR758, Molecular Probes) and Alexa568 (CentC, Roche). Following hybridization, slides were counterstained with DAPI (0.04 mg/ml) and ≥15 metaphase cells were evaluated per plant using a Zeiss Axio-Imager equipped with rhodamine, FITC, and DAPI filter sets (excitation BP 550/24, emission BP 605/70; excitation BP 470/40, emission: BP525/50; and excitation G 365, emission BP 445/50, respectively). Extrachromosomal signals were only considered to indicate autonomous MMCs if ≥70% of the images (*n* ≥ 15 cells analyzed) showed colocalization of the Alexa488 and Alexa568 signals within one nuclear diameter of the endogenous metaphase maize chromosomes. Grayscale images were captured in each panel, merged, and adjusted with pseudo-color using Zeiss AxioVision (Version 4.5) software; fluorescent signals from doubly labeled MMCs were detected in both the red and green channels.

### PCR and Southern blot analysis.

PCRs were carried out on genomic DNA isolated from young plants; qPCRs were performed in triplicate using a BioRad Chromo4 machine with TaqMan primers and probes (Sigma-Genosys). Amplification was achieved by incubating at 95 °C for 3 min, and 39 cycles of 95 °C for 15 s and 59 °C for 48 s, with a 1 s reduction per cycle. Copy number determinations were made by comparing qPCR signals from a control plasmid containing one copy of the maize *Adh1* gene and *DsRed* to the signals obtained from MMC-containing plants. For Southern blots, genomic DNA was isolated from young leaf tissue using a Nucleobond Plant Genomic DNA extraction kit (Clontech). Ten micrograms of DNA was digested with BglII (New England Biolabs), separated on a 0.7% agarose gel, vacuum transferred to a nylon membrane (Amersham BioSciences), and probed with a mixture of nonoverlapping pCHR758 fragments labeled with ^32^P (Rediprime II, Amersham BioSciences). Hybridization was performed overnight at 65 °C and blots were washed three times (15 min each) with 0.25× SSC, 0.1% SDS at 65 °C; signals were detected with a Storm phosphorimager.

### Sequencing and sequence analyses.

MMC1 was sequenced to an average of 30× coverage by shotgun sequencing (Lark Technologies) and 454 Technology (454 Life Sciences) and assembled with Phred/Phrap; a small gap was closed by primer walking, using direct dye-terminator cycling sequencing of MMC1. Quantitative dot blotting was used to calculate the total size of the CentC array. Briefly, two sets of blots, each containing samples in triplicate, were hybridized with CentC (CC) and vector specific (V) probes separately. Signals for each spot were captured with a Storm phosphorimager and CC/V ratios were calculated. Plasmids with the vector sequence and one, three, and eight copies of a cloned CentC repeat were used as standards. MMC1 assembly was verified by restriction mapping with panels of enzymes (BamHI, BmgBI, EcoRI and HindIII); this data was consistent with the calculated size of the CentC array. BLASTN (http://www.ncbi.nlm.nih.gov/BLAST/Blast.cgi) was used to assess sequence similarity, GENSCAN (http://genes.mit.edu/GENSCAN.html) to predict promoters and open reading frames, and repeat finder (http://tandem.bu.edu/trf/trf.basic.submit.html) to analyze CentC satellites.

### Statistical analyses.

The significance of MMC1 inheritance data was determined with a chi-square goodness-of-fit test. Differences from Mendelian segregation (based on a 1:1 segregation ratio in crosses to wild type or a 3:1 segregation ratio in self crosses or crosses to sibling plants) were considered significant at *p* < 0.05 (or a chi-square value greater than 3.84).

## Supporting Information

Figure S1 Metaphase Chromosome Spreads Labeled with FISH Probes Specific for the *DsRed* and *nptII* Gene Cassette (Green) or Centromere Sequences (Red) DNA is stained with DAPI (blue); autonomous MMCs (arrowheads). Size bar, 5 μm
(2.5 MB PDF)

### Accession Numbers

The National Center for Biotechnology Information (NCBI) GenBank database (http://www.ncbi.nlm.nih.gov/sites/entrez?db=Nucleotide&itool=toolbar) accession numbers for the sequences discussed in this paper are: Cent4, AF242891; CentA, AF078917; CentC, AY321491; contig carrying the *Arabidopsis* UBQ10 promoter, AL161503; CRM, AY129008; MZEHETRO, M35408; *nptII*-carrying pBSL97, U35136; S. cerevisiae YAT1 promoter, L28920; TR-1, AY083992; *Z. mays Adh1*, *X04049*; and Z. mays MMC1, EU053446.

## Abbreviations

BAC: bacterial artificial chromosome
CEN: centromere
FISH: fluorescence in situ hybridization
HAC: human artificial chromosome
MMC: maize minichromosome
qPCR: quantitative PCR
YAC: yeast artificial chromosome

## References

[pgen-0030179-b001] Herrera S (2004). Industrial biotechnology–a chance at redemption. Nat Biotech.

[pgen-0030179-b002] Lorence A, Verpoorte R (2004). Gene transfer and expression in plants. Methods Mol Biol.

[pgen-0030179-b003] Birch RG (1997). Plant transformation: problems and strategies for practical application. Annu Rev Plant Physiol Plant Mol Biol.

[pgen-0030179-b004] Walker D, Boerma HR, All J, Parrott W (2002). Combining *cry1Ac* with QTL alleles from PI 229358 to improve soybean resistance to lepidopteran pests. Molec Breeding.

[pgen-0030179-b005] Yin Z, Plader W, Malepszy S (2004). Transgene inheritance in plants. J Appl Genet.

[pgen-0030179-b006] Kumar S, Allen GC, Thompson WF (2006). Gene targeting in plants: fingers on the move. Trends Plant Sci.

[pgen-0030179-b007] Gilbertson L (2003). Cre-lox recombination: Cre-ative tools for plant biotechnology. Trends Biotech.

[pgen-0030179-b008] Hamilton CM, Frary A, Lewis C, Tanksley SD (1996). Stable transfer of intact high molecular weight DNA into plant chromosomes. Proc Natl Acad Sci U S A.

[pgen-0030179-b009] He RF, Wang Y, Shi Z, Ren X, Zhu L (2003). Construction of a genomic library of wild rice and *Agrobacterium*-mediated transformation of large insert DNA linked to BPH resistance locus. Gene.

[pgen-0030179-b010] Burke DT, Carle GF, Olson MV (1987). Cloning of large segments of exogenous DNA into yeast by means of artificial chromosome vectors. Science.

[pgen-0030179-b011] Clarke L, Carbon J (1980). Isolation of a yeast centromere and construction of functional small circular chromosomes. Nature.

[pgen-0030179-b012] Cottarel G, Shero JH, Hieter P, Hegemann JH (1989). A 125-base-pair CEN6 DNA fragment is sufficient for complete meiotic and mitotic centromere functions in Saccharomyces cerevisiae. Mol Cell Biol.

[pgen-0030179-b013] Larin Z, Monaco AP, Lehrach H (1991). Yeast artificial chromosome libraries containing large inserts from mouse and human DNA. Proc Natl Acad Sci U S A.

[pgen-0030179-b014] Kuroiwa Y, Tomizuka K, Shinohara T, Kazuki Y, Yoshida H, Ohguma A (2000). Manipulation of human minichromosomes to carry greater than megabase-sized chromosome inserts. Nat Biotech.

[pgen-0030179-b015] Ebersole TA, Ross A, Clark E, McGill N, Schindelhauer D (2000). Mammalian artificial chromosome formation from circular alphoid input DNA does not require telomere repeats. Human Mol Genet.

[pgen-0030179-b016] Schueler MG, Higgins AW, Rudd MK, Gustashaw K, Willard HF (2001). Genomic and genetic definitions of a functional human centromere. Science.

[pgen-0030179-b017] Harrington JJ, Van Bokkelen G, Mays RW, Gustashaw K, Willard HF (1997). Formation of de novo centromeres and construction of first-generation human artificial microchromosomes. Nat Genet.

[pgen-0030179-b018] Ikeno M, Grimes B, Okazaki T, Nakano M, Saitoh K (1998). Construction of YAC-based mammalian artificial chromosomes. Nat Biotech.

[pgen-0030179-b019] Tsuduki T, Nakano M, Yasuoka N, Yamazaki S, Okada T (2006). An artificially constructed de novo human chromosome behaves almost identically to its natural counterpart during metaphase and anaphase in living cells. Mol Cell Biol.

[pgen-0030179-b020] Grimes BR, Rhoades AA, Willard HF (2002). α-satellite DNA and vector composition influence rates of human artificial chromosome formation. Mol Ther.

[pgen-0030179-b021] Mejia JE, Alazami A, Willmott A, Marschall P, Levy E (2002). Efficiency of *de Novo* centromere formation in human artificial chromosomes. Genomics.

[pgen-0030179-b022] Ohzeki J, Nakano M, Okada T, Masumoto H (2002). CENP-B box is required for de novo centromere chromatin assembly on human alphoid DNA. J Cell Biol.

[pgen-0030179-b023] Okamoto Y, Nakano M, Ohzeki J, Larionov V, Masumoto H (2007). A minimal CENP-A core is required for nucleation and maintenance of a functional human centromere. EMBO J.

[pgen-0030179-b024] Meluh PB, Yang P, Glowczewski L, Koshland D, Smith MM (1998). Cse4p is a component of the core centromere of Saccharomyces cerevisiae. Cell.

[pgen-0030179-b025] Takahashi K, Chen ES, Yanagida M (2000). Requirement of Mis6 centromere connector for localizing a CENP-A-like protein in fission yeast. Science.

[pgen-0030179-b026] Malik HS, Henikoff S (2001). Adaptive evolution of Cid, a centromere-specific histone in Drosophila. Genetics.

[pgen-0030179-b027] Talbert PB, Masuelli R, Tyagi AP, Comai L, Henikoff S (2002). Centromeric localization and adaptive evolution of an Arabidopsis histone H3 variant. Plant Cell.

[pgen-0030179-b028] Zhong CX, Marshall JB, Topp C, Mroczek R, Kato A (2002). Centromeric retroelements and satellites interact with maize kinetochore protein CENH3. Plant Cell.

[pgen-0030179-b029] Palmer DK, O'Day K, Wener MH, Andrews BS, Margolis RL (1987). A 17-kD centromere protein (CENP-A) copurifies with nucleosome core particles and with histones. J Cell Biol.

[pgen-0030179-b030] Schatten G, Simerly C, Palmer DK, Margolis RL, Maul G (1988). Kinetochore appearance during meiosis, fertilization and mitosis in mouse oocytes and zygotes. Chromosoma.

[pgen-0030179-b031] Lo AW, Magliano DJ, Sibson MC, Kalitsis P, Craig JM (2001). A novel chromatin immunoprecipitation and array (CIA) analysis identifies a 460-kb CENP-A-binding neocentromere DNA. Genome Res.

[pgen-0030179-b032] Suzuki N, Nishii K, Okazaki T, Ikeno M (2006). Human artificial chromosomes constructed using the bottom-up strategy are stably maintained in mitosis and efficiently transmissible to progeny mice. J Biol Chem.

[pgen-0030179-b033] Ananiev EV, Phillips RL, Rines HW (1998). Chromosome-specific molecular organization of maize (Zea mays L.) centromeric regions. Proc Natl Acad Sci U S A.

[pgen-0030179-b034] Nagaki K, Song J, Stupar R, Parokonny A, Yuan Q (2003). Molecular and cytological analyses of large tracts of centromeric DNA reveal the structure and evolutionary dynamics of maize centromeres. Genetics.

[pgen-0030179-b035] Alfenito MR, Birchler JA (1993). Molecular characterization of a maize B chromosome centric sequence. Genetics.

[pgen-0030179-b036] Jin W, Lamb JC, Vega JM, Dawe RK, Birchler JA (2005). Molecular and functional dissection of the maize B chromosome centromere. Plant Cell.

[pgen-0030179-b037] Kaszas E, Kato A, Birchler JA (2002). Cytological and molecular analysis of centromere misdivision in maize. Genome.

[pgen-0030179-b038] Kato A, Zheng YZ, Auger DL, Phelps-Durr T, Bauer MJ (2005). Minichromosomes derived from the B chromosome of maize. Cytogenet Genome Res.

[pgen-0030179-b039] Phelps-Durr TL, Birchler JA (2004). An asymptotic determination of minimum centromere size for the maize B chromosome. Cytogenet Genome Res.

[pgen-0030179-b040] Yu W, Han F, Gao Z, Vega JM, Birchler JA (2007). Construction and behavior of engineered minichromosomes in maize. PNAS.

[pgen-0030179-b041] Kato A, Lamb JC, Birchler JA (2004). Chromosome painting using repetitive DNA sequences as probes for somatic chromosome identification in maize. Proc Natl Acad Sci U S A.

[pgen-0030179-b042] Grimes BR, Warburton PE, Farr CJ (2002). Chromosome engineering: prospects for gene therapy. Gene Therapy.

[pgen-0030179-b043] Dawe RK, Henikoff S (2006). Centromeres put epigenetics in the driver's seat. Trends Biochem Sci.

[pgen-0030179-b044] Co DO, Borowski AH, Leung JD, van der Kaa J, Hengst S (2000). Generation of transgenic mice and germline transmission of a mammalian artificial chromosome introduced into embryos by pronuclear microinjection. Chrom Res.

[pgen-0030179-b045] Ananiev EV, Riera-Lizarazu O, Rines HW, Phillips RL (1997). Oat-maize chromosome addition lines: A new system for mapping the maize genome. Proc Natl Acad Sci U S A.

[pgen-0030179-b046] Hall SE, Luo S, Hall AE, Preuss D (2005). Differential rates of local and global homogenization in centromere satellites from Arabidopsis relatives. Genetics.

[pgen-0030179-b047] Copenhaver GP, Nickel K, Kuromori T, Benito M-I, Kaul S (1999). Genetic definition and sequence analysis of *Arabidopsis* centromeres. Science.

[pgen-0030179-b048] Dong F, Miller JT, Jackson SA, Wang G-L, Ronald PC (1998). Rice (Oryza sativa) centromeric regions consist of complex DNA. Proc Natl Acad Sci U S A.

[pgen-0030179-b049] Kumar A, Bennetzen JL (1999). Plant retrotransposons. Annu Rev Genet.

[pgen-0030179-b050] Volpe T, Schramke V, Hamilton GL, White SA, Teng G (2003). RNA interference is required for normal centromere function in fission yeast. Chrom Res.

[pgen-0030179-b051] May BP, Lippman ZB, Fang Y, Spector DL, Martienssen RA (2005). Differential regulation of strand-specific transcripts from *Arabidopsis* centromere satellite repeats. PLoS Genet.

[pgen-0030179-b052] Topp CN, Zhong CX, Dawe RK (2004). Centromere-encoded RNAs are integral components of the maize kinetochore. Proc Natl Acad Sci U S A.

[pgen-0030179-b053] Talbert PB, Henikoff S (2006). Spreading of silent chromatin: inaction at a distance. Nat Rev Genet.

[pgen-0030179-b054] Einset J (1943). Chromosome length in relation to transmission frequency of maize trisomes. Genetics.

[pgen-0030179-b055] Telenius H, Szeles A, Kereso J, Csonka E, Praznovszky T (1999). Stability of a functional murine satellite DNA-based artificial chromosome across mammalian species. Chrom Res.

[pgen-0030179-b056] McClintock B (1938). The production of homozygous deficient tissues with mutant characteristics by means of the aberrant mitotic behavior of ring-shaped chromosomes. Genetics.

[pgen-0030179-b057] Runge KW, Zakian VA (1993). Saccharomyces cerevisiae linear chromosome stability (*lcs*) mutants increase the loss rate of artificial and natural linear chromosomes. Chromosoma.

[pgen-0030179-b058] Kaszas E, Birchler JA (1998). Meiotic transmission rates correlate with physical features of rearranged centromeres in maize. Genetics.

[pgen-0030179-b059] Dawe RK, Hiatt EN (2004). Plant neocentromeres: fast, focused, and driven. Chrom Res.

[pgen-0030179-b060] Hawley RS, Theurkauf WE (1993). Requiem for distributive segregation: achiasmate segregation in *Drosophila* females. Trends Genet.

[pgen-0030179-b061] Mehta S, Yang X-M, Jayaram M, Velmurugan S (2005). A novel role for the mitotic spindle during DNA segregation in yeast: promoting 2 μm plasmid-cohesin association. Molec Cell Biol.

[pgen-0030179-b062] Amor DJ, Choo KHA (2002). Neocentromeres: role in human disease, evolution, and centromere study. Am J Hum Genet.

[pgen-0030179-b063] Heun P, Erhardt S, Blower MD, Weiss S, Skora AD (2006). Mislocalization of the *Drosophila* centromere-specific histone CID promotes formation of functional ectopic kinetochores. Dev Cell.

[pgen-0030179-b064] Wu J, Yamagata H, Hayashi-Tsugane M, Hijishita S, Fujisawa M (2004). Composition and structure of the centromeric region of rice Chromosome 8. Plant Cell.

[pgen-0030179-b065] Frame BR, Zhang H, Cocciolone SM, Sidorenko LV, Dietrich CR (2000). Production of transgenic maize from bombarded type II callus: effect of gold particle size and callus morphology on transformation efficiency. In Vitro Cell Dev Biol Plant.

[pgen-0030179-b066] Kato A (1999). Air drying method using nitrous oxide for chromosome counting in maize. Biotech Histochem.

