# Supplementary material for: Meiotic Transmission of an In Vitro–Assembled Autonomous Maize Minichromosome
Source: PLoS Genet. 2007 Oct 19;3(10):e179. doi: 10.1371/journal.pgen.0030179 (PMC2041994; doi:10.1371/journal.pgen.0030179)
Supplement: Supplementary file 1 [file pgen.0030179.sg001.pdf]

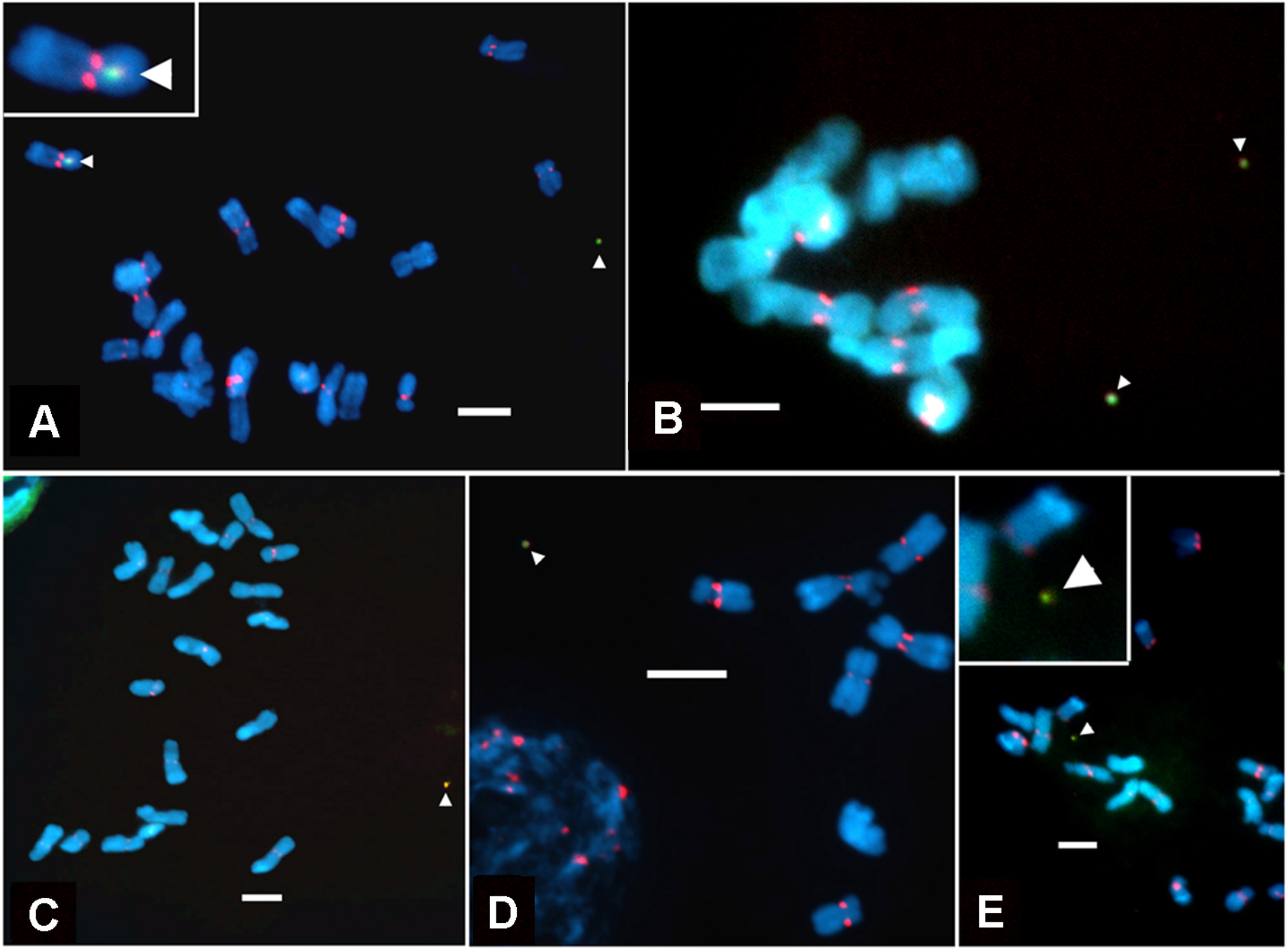

Figure S1
